# Mental health consequences of infections by coronaviruses including severe acute respiratory syndrome coronavirus 2 (SARS‐CoV‐2)

**DOI:** 10.1002/brb3.1901

**Published:** 2020-12-13

**Authors:** Suliman Khan, Rabeea Siddique, Wang Xiaoyan, Ruiyi Zhang, Ghulam Nabi, Muhammad Sohail Afzal, Jianbo Liu, Mengzhou Xue

**Affiliations:** ^1^ The Department of Cerebrovascular Diseases The Second Affiliated Hospital of Zhengzhou University Zhengzhou China; ^2^ Henan Medical Key Laboratory of Translational Cerebrovascular Diseases Zhengzhou China; ^3^ Child Health Department, Maternal and Child Health Hospital of Hubei Province, Tongji Medical College Huazhong University of Science and Technology Wuhan China; ^4^ Key Laboratory of Animal Physiology, Biochemistry and Molecular Biology of Hebei Province College of Life Sciences Hebei Normal University Shijiazhuang China; ^5^ Department of Life Sciences School of Science University of Management and Technology (UMT) Lahore Pakistan; ^6^ Deparmtent of respiratory diseases The Second Affiliated Hospital of Zhengzhou University Zhengzhou China

**Keywords:** cerebrovascular conditions, COVID‐19, psychological health, spread

## Abstract

**Background:**

Anxiety and stress like mental illnesses are the common outcomes of viral epidemics and pandemics. Novel coronavirus disease 2019 (COVID‐19) outbreak caused by the severe acute respiratory syndrome coronavirus virus 2 (SARS‐CoV‐2) was first reported in Wuhan, China, and then spread all over the world in a short time.

**Objectives:**

To highlight and discuss the impact of COVID‐19 pandemic on mental or psychological health.

**Method:**

Literature search and collection of the information were performed using PubMed, the reports from the World health organization, and the Center for disease control and prevention.

**Results:**

COVID‐19 infection has already been declared as a global pandemic, which in association with infodemic has increased the risk of psychiatric/psychological disorders. A large population of the world is prone to develop anxiety, depressive disorders, and other mental abnormalities. Therefore, timely psychological interventions and preventive strategies are required. Moreover, the infection has been reported to be linked with cerebrovascular conditions; therefore, patients with underlying cerebrovascular diseases should be given attention.

**Conclusion:**

COVID‐19‐mediated mental health complications and cerebrovascular conditions may cause a huge burden on healthcare communities in the future. Therefore, timely intervention and the development or application of preventive strategies are required to decrease the risk of neurological consequences.

## Graphical abstract

Mental illnesses are the common outcomes of the viral outbreaks. Novel coronavirus disease 2019 (COVID‐19) pandemic has increased the risk psychiatric disorders. A large population of the world is prone to develop anxiety, depressive disorders, and other mental abnormalities. Therefore, timely psychological interventions and preventive strategies are required.

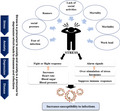

## 1 INTRODUCTION

The mental health consequences are not only associated with deadly viral infections such as Ebola (Van Bortel et al., 2016) but also associated with the common viral infections such as herpes (Coughlin, 2012). However, these consequences are become severe in response to epidemics or pandemics of rapidly spreading viruses, which have high rates of morbidity and mortality, and lack diagnostics and treatment options (Khan, Siddique, Li, et al., 2020). These characteristics are most suited with coronaviruses which can cause deadly diseases in humans. Besides the physical health complications, human coronaviruses, most prominently SARS‐CoV‐2, can damage the mental health of individuals during the outbreak (COVID‐19) (Khan, Siddique, Ali, et al., 2020). The SARS‐CoV‐2 primarily infects the lungs; however, it may also target the brain (Li et al., 2020), which contains ACE2 receptors (Baig et al., 2020). Patients with underlying cerebrovascular disease can be more sensitive to the infection and can develop severe symptoms (Mao et al., 2020). In this article, we have highlighted how human coronaviruses can affect the mental health of a population during an outbreak.

## 2 METHODS

### 2.1 Strategy to address the objective

The objective of the current study was addressed using the evidence‐based information related to the impact of COVID‐19 on psychiatric or mental health.

### 2.2 Search strategy

The electronic database PubMed was searched to identify the relevant studies using the search terms “COVID‐19, SARS‐COV, Mental health and COVID‐19, psychological impacts of COVID‐19, COVID‐19 and cerebrovascular diseases/brain diseases, COVID‐19 and brain, transmission and spread of COVID‐19, and entry of SARS‐CoV‐2 to human cells.” Original research articles, review articles including narrative and systematic reviews, and case reports were included in the current study. Moreover, details related to COVID‐19 infection and mental health or spread of SARS‐CoV‐2 were obtained from reports published by the World Health Organization and Centers for Diseases Control and Prevention. The articles published in English were considered for this study; however, no limit was applied for searching articles.

## 3 RESULTS

### 3.1 Severe acute respiratory syndrome (SARS) outbreak

The SARS‐CoV‐2 transmitted from bats to civet cats and then to humans has caused the SARS outbreak in Guangdong province China during 2002. This outbreak later affected 29 countries and resulted in 8,422 victims with 916 fatal cases (Lin et al., 2007). It was a unique outbreak in its rapidity of transmission and its concentration in healthcare settings, where several from a large number of infected healthcare workers died (Lin et al., 2007). With a rapid person‐to‐person transmission *via* respiratory droplets and high mortality/morbidity rates, the SARS outbreak fostered negative effects on mental health, prominently in medical staff, who were diagnosed with distress, hopelessness, anxiety, and feeling of incapability to deal with infected patients (Hawryluck et al., 2004; Lin et al., 2007; Zumla et al., 2016).

### 3.2 Middle East respiratory syndrome (MERS) outbreak

The MERS outbreak caused by the Middle East respiratory syndrome coronavirus (MERS‐CoV) was reported after a cluster of nosocomial cases in Jordan during April 2012 (Hijawi et al., 2013). The spread of MERS‐CoV continued beyond the Middle East and several other countries. Until 2020, overall 2,468 cases and 851 fatalities were reported globally (Sheahan et al., 2020). The fear of infection and higher fatality in MERS outbreak induced psychological distress in people and severe mental illness in nurses and doctors who worked at the front line in isolation areas, intensive care units (ICUs), and emergency rooms (Bukhari et al., 2016; Lee et al., 2018). The MERS outbreak created widespread fear and panic among healthcare workers (Almutairi et al., 2018). It has been reported that infecting mice with low inoculum doses of MERS‐CoV resulted in the detection only in the brain, but not in the lung, suggesting that the virus may primarily affect the central nervous system (CNS). This observation can be related to mortality in infected individuals (Li et al., 2020).

### 3.3 COVID‐19 outbreak

When SARS occurred, the awareness regarding mental health was low and no psychological guidelines existed for isolation situations and large‐scale quarantine during the epidemic or pandemic period (Lee et al., 2018). However, the numbers of infected individuals were low to be readily handled by healthcare authorities and sufficient healthcare services were available. In contrast, the efficiently spreading COVID‐19 infection has infected large populations (Khan, Ali, et al., 2020) and caused millions of morbidities and near a million mortalities worldwide. To curb the risks of epidemic and pandemic, large populations have been exposed to the preventive actions imposed by the regulatory authorities across the different countries of the world including China, the United States of America, the United Kingdom, France, Italy, Canada, India, and Iran (Khan, Siddique, Ali, et al., 2020). Due to asymptomatic spread (Rothe et al., 2020) and human‐to‐human transmission of the virus, the numbers of confirmed cases unexpectedly increased, that increased a huge burden on the healthcare workers and, ultimately, increases the risks of developing mental illnesses (Khan, Siddique, Bai, et al., 2020; Khan, Siddique, Li, et al., 2020, 2020).

### 3.4 Transmission and entry mechanisms

SARS‐CoV‐2 has been reported to be transmitted to humans from the market selling wild animals in Wuhan, which started to spread rapidly because of having a high “human‐to‐human” transmission potential (Cui et al., 2019). Although the mechanisms of infectiousness are not clear for SARS‐CoV‐2, however, they most likely enter human cells through the ACE2 receptor (Zhou et al., 2020). Moreover, SARS‐CoV‐2 has much more similarity with SARS‐CoV (Alagaili et al., 2014; Guan et al., 2003) having a highly conserved protease enzyme with a 96% similarity to that of SARS‐CoV (Provincial et al., 2020). These similarities suggest that bats could be the source of origin, while a yet unknown animal might be an intermediate host that had facilitated the transmission of SARS‐CoV‐2 into humans (Wu et al., 2020; Zhou et al., 2020). The transmission of SARS‐CoV‐2 from an infected to healthy individuals occurs *via* respiratory droplets during coughing or sneezing or aerosol formation (Phan et al., 2020) and through the fecal–oral route. Moreover, asymptomatic transmission has also been reported by several studies (Rothe et al., 2020).

A virus entry to the host cell comprises a series of fundamental interactions: binding to the host cell using cellular receptors, the fusion of the envelope with a cellular membrane, and forking over its genetic material inside the cell (Khan, Liu, et al., 2020). The delivery of viral genetic material or nucleic acids into the host cell depends upon the viral efficiency/specificity for binding to receptors, proteolytic activation, and efficiency for endocytosis (Boulant et al., 2015; White & Whittaker, 2016). The entry process of coronaviruses occurs by demonstrating a great degree of plasticity that allows the entry through the plasma membrane or endocytic pathway (Belouzard et al., 2009). This entry in the case of SARS‐CoV‐2 is regulated by glycosylated spike fusion protein and ACE2. The capability of S proteins in the aspects of structural rearrangement plays an important role in the fusion process of both viral and host cell membranes (Li, 2016). The fusion process is sparked off by binding of the S1 to ACE2, and the process is also linked with receptor accessibility determined by conformational movements (hinge‐like) of RBD of S1 (Gui et al., 2017). The replication–transcription complex (RTC) is organized in double‐membrane vesicles after the entrance of the virus, and thus, transcription of polyproteins is initiated (Cascella et al., 2020), which plays a major role in the pathogenicity of SARS‐CoV‐2 (Lei et al., 2018).

### 3.5 Impact of COVID‐19 outbreak on mental health

**Figure 1:**
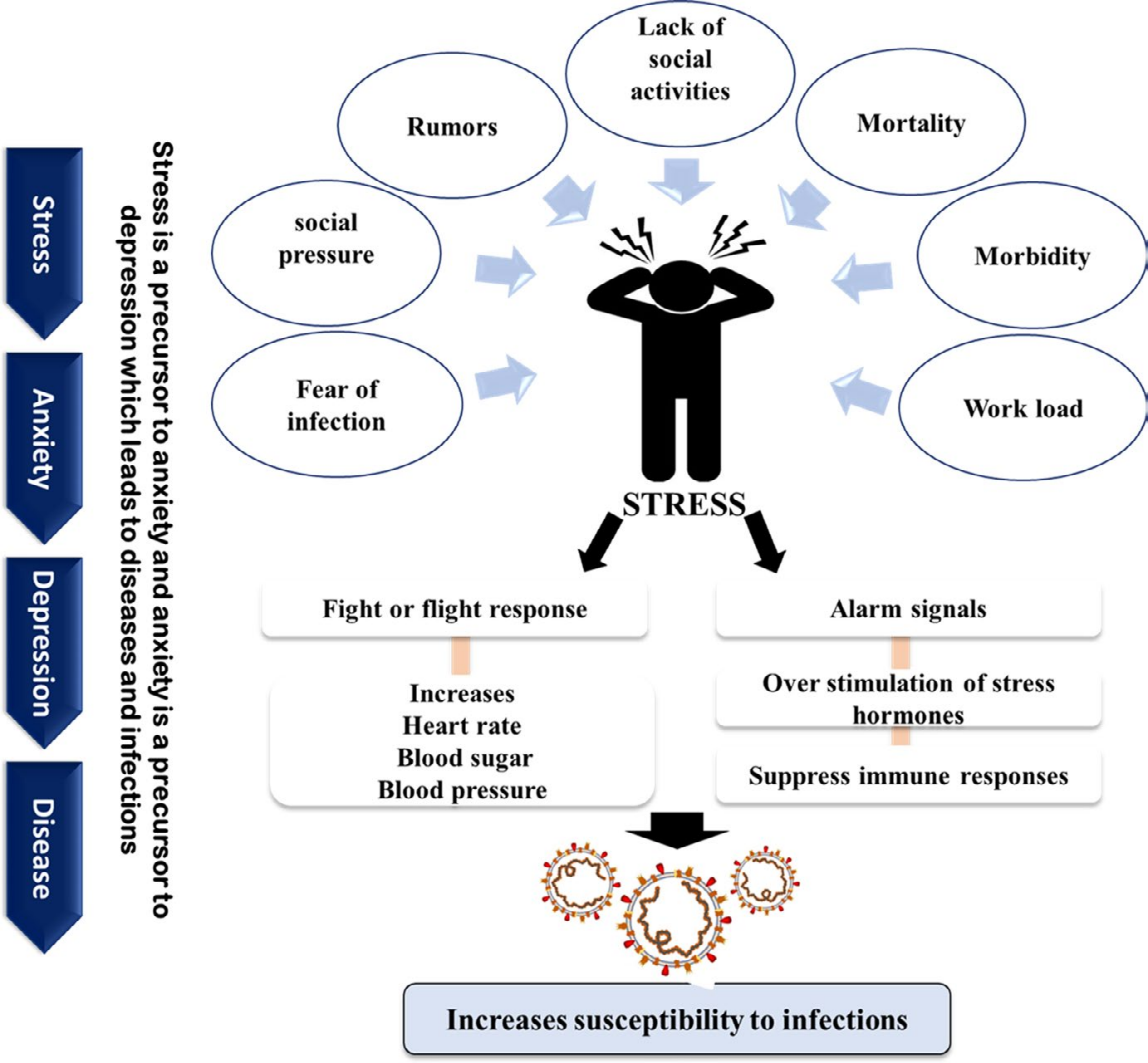
Different risk factors associated with novel coronavirus outcome induce stress that further increase the risk of infectiousness

COVID‐19 outbreak is affecting physical health and mental health; however, the primary attention is given to physical health. Like SARS and MERS outbreaks, the healthcare workers are at the highest risk of contracting the infection in the COVID‐19 outbreak. A large number of medical staff have been infected, and further infection in medical workers may create an alarming situation for healthcare authorities across the world (Khan, Nabi, et al., 2020). Thus, to deal with the rapid increase in the number of infected individuals, doctors and nurses are forced to work for longer hours. This situation affects the working efficiencies of medical and clinical workers and thus can increase the risk of fatality among infected individuals. Overall, the fear of being infected due to close contact with infected patients, prolonged working schedules without proper rest, and disturbed wake and sleep routines has increased the risk of stress and anxiety in the remaining health workers, who are working at the front line (Khan, Nabi, et al., 2020). Mental illnesses have been reported to alter immunity, thus increasing the vulnerability to diseases (Chiang et al., 2019) as depicted in Figure 1. It might be one of the risk factors for higher COVID‐19‐mediated morbidity among medical staff and clinical workers.

### 3.6 Psychological interventions

During the start few months of the COVID‐19 outbreak, the Institute of Mental Health in association with the Chinese Medical and Psychological Disease Clinical Medicine Research Center developed a psychological intervention plan. Unfortunately, these services encountered obstacles such that the majority of medical and clinical staff were reluctant to psychological interventions provided to them (Khan, Siddique, Bai, et al., 2020; Khan, Siddique, Li, Ali, et al., 2020; Khan, Siddique, Li, Xue, et al., 2020; Khan, Siddique, Shereen, et al., 2020). Effective psychological counseling addressing the major issues with medical staff including fear of bringing the virus to their homes, difficulty in dealing with patients unwilling to cooperate with medical measures due to panic, shortage of protective equipment, and lack of confidence to face the critically ill patients.

The researchers have started a wide range of surveys related to mental health which target different populations including medical staff, patients with COVID‐19, students, and the general population in different provinces, municipalities, and autonomous regions. These findings may help health authorities to allocate health resources for individuals with mental health problems. In addition, online psychological counseling services have been tested during the outbreak for medical staff and infected patients (Liu et al., 2020).

Psychological intervention can be carried out by providing psychological therapies. Resilience is the ability to sustain psychological well‐being after going through a stressful condition. Resilience should be considered in the current pandemic of COVID‐19 in order to prevent stress and mental impairment (Evers et al., 2011; Khan, Siddique, Li, Ali, et al., 2020). In addition to resilience, psychological debriefing may also help in the alleviation of acute distress and the prevention of chronic depression and anxiety. More importantly, psychological debriefing will be helpful for the people who are likely to develop psychotic irrespective of their severity. The instructors should educate the public that psychological problems are normal responses to the COVID‐19 outbreak. Furthermore, cognitive behavior therapy can also be used to combat psychiatric symptoms caused by COVID‐19 disease (Khan, Siddique, Li, Ali, et al., 2020).

#### 3.6.1 The inefficiency of psychological intervention strategies

A large fraction of people cannot get benefit from the online mental health services due to limited access to the internet, computers, and smartphones (Yang et al., 2020). Foreign nationals, particularly migrant workers, encounter barriers in accessing health services, necessary commodities, and receiving accurate information regarding the spread and protection from COVID‐19 infection. The only available online services may not work as most of the services are provided in languages other than these individuals can understand. Thus, they require special attention not only from the healthcare services providers but also from the local people in communities and their employers. Moreover, long‐term quarantine, lost income, no access to masks and preventive measures, fear of infection, and fear of no cooperation, if contracted the infection, further increase the severity of mental illness (Liem et al., 2020). Providing these populations with effective mental health services is challenging in the current scenario of the COVID‐19 pandemic.

### 3.7 Circadian disruption and mental diseases in children

Well‐regulated daily routines are inevitable to maintain normal rhythms; however, in the current mass quarantines with limited to activities in the natural environment, millions of people have been restricted to stay indoors, where they mostly spend time using electronic devices. These people can experience exposure to irregular light–dark cycles, disturbed sleep–wake behaviors, and eating routines, which may dysregulate normal biological rhythms and mood (Khan et al., 2018). Generally, children and young adults are vulnerable to disruptions in circadian rhythm and thus can develop severe mental illnesses (Zitting et al., 2018). To mitigate the risks of such complications, children and young kids should be given full attention engaging them in healthy activities after the pandemic is over. However, the government may enforce the working population to work overtime; therefore, the authorities should come up with effective plans.

### 3.8 COVID‐19 infection and neurological conditions

Individuals with underlying medical conditions such as diabetes and hypertension are at higher risk of contracting infection (Huang et al., 2020). A higher fatality rate was observed in individuals with underlying diabetes mellitus and chronic lung disease (Wu & McGoogan, 2020), cardiopulmonary diseases, hypertension (Wu & McGoogan, 2020), and lung cancer (Luo et al., 2020). Luo et al. (2020) reported that a large number of COVID‐19 patients with underlying lung cancer required critical care, and nearly a quarter of these patients died (Luo et al., 2020).

The SARS‐CoV‐2 mainly targets the lungs but could affect the heart and brain's health (Li et al., 2020). Previous studies have reported that in transgenic mice, SARS‐CoV could enter the brain possibly through the olfactory nerves and spread to the thalamus, brainstem, and some other brain regions (Li et al., 2020). Similarly, COVID‐19 causes the loss of smell and taste (Baig et al., 2020; Wang et al., 2020), suggesting that the virus can affect neurons (olfactory neurons) in the brain. Moreover, COVID‐19 may also affect the brainstem, thereby causing defects in the respiratory center of the nervous system.

Some of the COVID‐19 patients were found with neurological problems such as headaches and nausea (Wang et al., 2020). Mao et al. (2020) reported that a higher ratio of severe patients displayed neurologic manifestations including impaired consciousness and acute cerebrovascular diseases. Investigations have revealed that the brain expresses ACE2 receptors over glial cells and neurons, suggesting that these are the potential targets for COVID‐19 infection (Baig et al., 2020). The glial cells are the key player in stroke and cerebrovascular disorders (Campbell et al., 2019; Morris et al., 2019), suggesting that the infection of glial cells by COVID‐19 may be the possible reasons for higher risk for cerebrovascular problems. However, investigations are required to determine the underlying mechanism for COVID‐19 infection and the development of cerebrovascular diseases.

## 4 CONCLUSIONS

The emergence of COVID‐19 and the menace of imminent endemics have brought into the forefront the urgent need to prepare for the consequences of further epidemics and pandemics. Ignoring the profound importance of the brain's health and psychological health, ensued by the COVID‐19 infection, and restricted social activities and culminating into stress and anxiety. Patients with underlying neurological diseases such as stroke should be given proper attention. To cope with the psychological consequences of the outbreak, clinical psychologists and psychiatrists should come forward and provide their services that could help the medical staff and clinical workers to work efficiently and the general public to stay healthy. The COVID‐19 outbreak will eventually end, but it could cost thousands of lives and millions with severe mental problems. Dealing properly with this critical situation, the top of the government's agenda should be the development of a huge network of clinical psychologists and psychiatrists to assist the public, doctors, medical assistants, technicians, and nurses working at the front line.

## CONFLICT OF INTEREST

The authors declare that they have no conflict of interest.

## AUTHOR'S CONTRIBUTION

Suliman Khan contributed to the conception, organization, execution, and manuscript drafting. Suliman Khan, Rabeea Siddique, Wang Xiaoyan, Ruiyi Zhang, and Ghulam Nabi contributed to the revision. Mengzhou Xue and Jianbo Liu supervised the study. All the authors approved the final version of the manuscript.

### Peer Review

The peer review history for this article is available at https://publons.com/publon/10.1002/brb3.1901.
